# Increasing Divergent Thinking Capabilities With Music-Feedback Exercise

**DOI:** 10.3389/fpsyg.2020.578979

**Published:** 2020-11-19

**Authors:** Thomas Hans Fritz, Max Archibald Montgomery, Eric Busch, Lydia Schneider, Arno Villringer

**Affiliations:** ^1^Max Planck Institute for Human Cognitive and Brain Sciences, Leipzig, Germany; ^2^Institute for Psychoacoustics and Electronic Music (IPEM), Ghent, Belgium

**Keywords:** athlete performance, divergent thinking, creativity, Jymmin, musical agency, neurology of music, team sports, athlete performance enhancement

## Abstract

Divergent thinking is an essential aspect of creativity and has been shown to be affected both by music and physical exercise. While it has been shown that making music and physical exercise can be beneficial for Divergent Thinking in isolation, it is unclear whether the effects can be combined. The present experiment investigated the relation of physical exertion and being in control of music on Divergent Thinking and the possibility of an interaction effect. Seventy-seven predominantly young, German participants were tested with measurements of Divergent Thinking collected after either (1) physical exercise with music listening, (2) making music with a knob setup without physical effort (music control only), or (3) making physical exercise with musical feedback (Jymmin™). Results showed greater increases in Divergent Thinking scores following music-feedback exercise compared to conditions of physical exercise with music listening and music control only. The data thus demonstrate that making music part of a physical exercise routine more strongly leads to the benefit of increased creative capacities, which we argue will be beneficial for athletes to prepare for certain types of competition/performance and as part of regeneration training.

## Introduction

Mental blocks to creativity are often a challenge for workers in the creative industries. It has been argued that, for example, expectations of external judgment would lead to an increase in scrutiny of ideas, leading to rejection before the idea is allowed to reach full fruition, hence leading to a net decrease in creativity (Amabile, 1979). The movie depiction of overcoming such a mental block often involves the use of intoxicating substances. Healthier approaches involve physical exercise (Oppezzo and Schwartz, 2014) and making music (Gibson et al., 2009). A wealth of literature highlights a number of benefits of combining exercise with music (we point curious readers to the excellent summary by Karageorghis, 2017, for a thorough review). How combining exercise and music to increase creativity is still poorly understood.

An underexplored topic in the research of the benefits of music is the importance of experiencing control of the music. This idea has been indirectly approached in other studies (Fritz et al., 2013a,b, 2016, 2015) where control of the music is termed musical agency. It was observed in these previous studies that musical agency alone was not the driver of the observed cognitive and physiological effects but that it was the combination of musical control and physical exertion that made the difference. Here, we addressed the question whether musical control would impact creativity and if musical control needed to be coupled with physical exercise in order to be more effective. In the present study, we focused on Divergent Thinking, a useful measure of creativity.

Divergent Thinking is a construct defined as the capacity to generate novel solutions to a given problem and has been shown to be a crucial component in the process of idea generation (Guilford, 1956; Vincent et al., 2002). A change in the level of Divergent Thinking ability can be successfully quantified using standardized scoring methods (Runco et al., 1987) where the solutions generated during the test are assessed on usefulness and novelty (Runco and Jaeger, 2012).

Divergent Thinking has been shown to increase when participants perform physical activities (Colzato et al., 2013) including walking (Oppezzo and Schwartz, 2014) and dancing (Gondola, 1987; Blanchette et al., 2005). Creativity has also been positively linked with making music (Kleinmintz et al., 2014) and listening to music (Ritter and Ferguson, 2017), suggesting that music may be especially stimulating for creativity. One study investigated the effects of more specific types of musical engagement further and found novel generation of music to increase scores more than playing learned melodies (Lewis and Lovatt, 2013). Because the comparison in Lewis and Lovatt (2013) was musical improvisation to non-improvisation, we would expect that the beneficial effect of music on Divergent Thinking depends on the nature of the musical task and that increased musical control would yield increased Divergent Thinking scores compared to just music listening.

Here, we present a framework for increasing Divergent Thinking capabilities as part of a physical exercise by using a musical feedback exercise called Jymmin^TM^ (Fritz et al., 2013a). By using this method, participants are able to generate music as a by-product of exercising with traditional training machines such as a lat pull-down, stepper, ab-trainer, and other machines commonly used in weight training, as well as with gymnastics movements. Jymmin^TM^ has previously been shown to have certain cognitive effects such as heightening mood (Fritz et al., 2013b), reducing perceived exertion (Fritz et al., 2013a), increasing the aesthetic evaluation of music (Fritz et al., 2016), increasing perception of self-efficacy (Fritz et al., 2015), and decreasing the perception of pain (Fritz et al., 2018). A physiological effect at muscle level that seems to relate to greater muscle efficiency/muscle relaxation has also been reported (Fritz et al., 2013a). Music-feedback exercise may be useful to healthy populations as a way to make strenuous exercise more palatable and motivating or by groups with motor/cognitive deficits to give a clear feedback about the exercise.

Using a repeated measures design with one factor and three levels, we here investigated both the impact of musical control and of exercise on Divergent Thinking as measured by a Guilford’s Alternative Uses Task. We also surveyed participants on mood, subjective creativity, and perceived control of the music. We hypothesized that participants in the “music-feedback exercise” condition would achieve significantly higher Divergent Thinking scores compared to the “music control only” and “physical exercise with music listening” conditions due to a positive interaction between musical control and physical exertion due to exercise. We also expected participants in the music control and physical exercise with music listening conditions to elicit elevated Divergent Thinking scores but to a lesser degree than in the music-feedback exercise condition.

## Materials and Methods

### Participants

Seventy-nine German-speaking participants, 18 male and 44 female, were recruited from a participant archive compiled by the Max Planck Institute for Cognitive and Brain Sciences in Leipzig. Seventeen participants were excluded due to unintelligible handwriting. The final sample was 62 participants of which 4 were left-handed, 18 were male and 44 were female, aged between 19 and 52 (M = 26.20, SD = 5.82). Professional musicians and athletes were also excluded, along with participants who had already taken part in other studies with the same music-feedback paradigm. Ethical approval was granted by the University of Leipzig ethics committee. All participants provided informed consent prior to commencing the study and were compensated for their time.

### Materials

#### Experimental Setup

The experiment was conducted in a large, well-ventilated, temperature-controlled room. The physical exercise setup consisted of one abdominal trainer, one stepper, and one lat pull-down machine (see online Supplementary Material for a full description). In the music-feedback exercise condition, each machine was modified with a movement sensor, which continually transmitted its position to a computer that modified musical material to create a music feedback (Fritz et al., 2013b) based on the current position of the sensor. Each sensor continually transmitted its position to an embedded system, which “translated” the positional value of the sensor into its corresponding musical output. This effectively transformed each machine into an analog for a musical instrument. Each machine controlled a musical dimension with trigger points corresponding to different position values of the sensors on the training machines, where sounds and loops were combined to create a coherent musical output. The musical output from each machine was composed such that it combined well with the others (e.g., same tempo and tonality; for a video demonstration, see www.jymmin.com). The musical piece to be produced by the upgraded training machines was composed by a student of electroacoustic music composition (EB). It included harmonically and rhythmically complex components and is best described as experimental electronic music (see online Supplementary Material for audio example). This complex music was chosen to introduce a variability in how different participants would feel in control of the music and to ensure that the musical experience was novel to all the participants.

For the music control only condition, we employed a knob setup that allowed participants to modify the music software similarly to how it could be modified with training machines in the music-feedback group but without physical exertion.

#### Outcome Measures

Participants were asked to fill out three questionnaires: first is a questionnaire assessing demographic information of age, gender, and handedness; second is a Guilford’s Alternative Uses Task (Guilford, 1967) in which participants were given a piece of paper and asked to name as many uses as possible for either a bottle or a brick in order to control for the confounding effect of item order presentation. The task was counterbalanced with half the participants conducting the brick version of the task as baseline and the bottle postintervention. The other half took the test with the bottle as a baseline measure and the brick as a postintervention measure. Participants were given 2 min (timed with a stopwatch) to fill in their answers. The test was taken in the experimental groups. No talking was allowed during the test. Third is a self-report questionnaire assessing perceived musical control, mood, feeling in touch with the music, and perceived creativity. The questionnaire asked participants to assess how much they agreed to a series of statements such as “I felt in touch with the music” on a visual analog scale of 0–100.

#### Procedure

Participants conducted the experiment in groups of 3. Each group was randomly assigned to an experimental condition. Participants were randomly assigned to a training machine in the physical exercise with music listening and music-feedback exercise conditions and a set of musical sounds in the music control only condition. The experiment had a duration of 45 min including briefing, intervention, assessments, and debrief. In cases where one participant did not show up, an experimenter would take their place to ensure that the musical output stayed consistent across groups. Each group was randomly assigned to one of three conditions:

In the “music-feedback exercise” group, participants performed a task where musical control was combined with physical exercise (Jymmin^TM^). The “music control only” group employed a knob setup to make music (without physical exertion). The “physical exercise with music listening” group performed exercise while also listening to a musical output that had been produced by a previous “music-feedback exercise” group.

### Timeline

Participants were asked to fill out a Guilford’s Alternative Uses Task employing either a bottle or a brick. Participants were then asked to perform for 10 min with their assigned setup. When the intervention was completed, participants were given another Guilford’s Alternative Uses Task. Participants were then asked to fill out a questionnaire relating to their experience of the experiment, including measures of perceived musical control and creativity.

### Data Management

Two independent raters, blind to the experimental hypothesis, were trained to score the Guilford’s Alternative Uses Tasks. Both raters were native German speakers and completed the scoring independently. Questionnaires were scored with a composite score based on originality, fluency, flexibility, and elaboration. Each answer was worth 1 point, with additional points being given according to the suggestions set by Guilford, 1967. Questionnaire responses that were deemed “unintelligible” by one rater were excluded from further analysis. The raters disagreed on the scoring of four participants with respect to whether to award points for originality. The disagreements were resolved in concert with the two reviewers and one of the authors (MM), who was blinded to participant IDs and condition and who acted as an arbiter applying the rating guidelines. Divergent Thinking scores were summarized and computed to a single score that represented the net change in creativity from the pretest to the posttest. This was achieved by adding the four subscores together for both the pretest and the posttest Divergent Thinking tasks individually. The total score of the pretest was then subtracted from the posttest scores. A Kolmogorov–Smirnov test of normality was computed to assess the normal distributions of the Divergent Thinking score (posttest minus pretest). Analysis revealed the groups to be normally distributed in the Divergent Thinking score change statistic, *D*(65) = 0.085, *p* = 0.200. Perceived creativity D(0.143), *p* = 0.004; musical control, *D*(0.132), *p* = 0.011, and mood, *D*(0.188), *p* < 0.001 were found to violate assumptions of normality. We therefore proceeded with a parametric analysis of the Divergent Thinking scores and non-parametric analyses for the mood and self-report questionnaire items. The data was analyzed with SPSS 27.

## Results

A univariate analysis of variance was conducted assessing the hypothesis that creativity scores would increase in the music-feedback exercise condition compared to the other two conditions. Age, gender, and handedness were added as covariates to control for individual differences. Analysis revealed a significant effect of condition *F*(2,59) = 5, *p* = 0.002.

**FIGURE 1:**
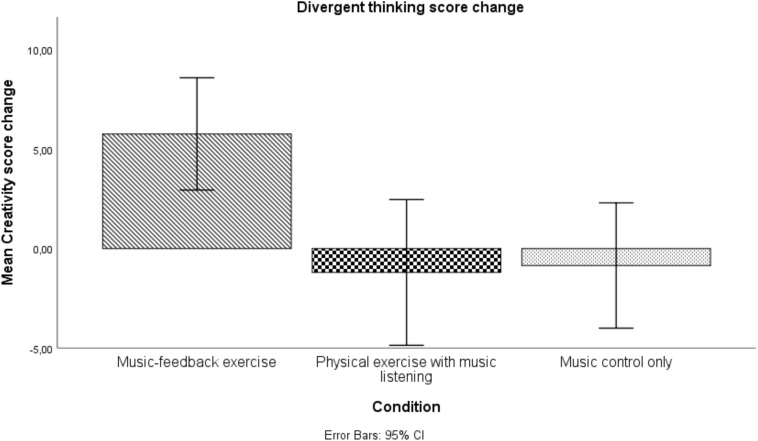
Mean change in score from pretest to posttest on a Guilford’s Alternative Uses task. Error bars represent the 95% confidence interval.

Multiple comparisons using the Bonferroni correction revealed a significant difference between the music-feedback exercise group (M = 5.77, SD = 6.37) and the two conditions of physical exercise with music listening (M = -−0.20, SD = 7.83; *p* = 0.00) and music control only (M = −0.85, SD = 6.73; *p* = 0.01). No significant difference was found between physical exercise with music listening and music control only. Results are detailed in Figure 1.

A separate Kruskal–Wallis test was also conducted, assessing the exploratory measures of mood, perceived musical control, self-rated creativity, and “feeling in touch with the music.” Age, gender, and handedness were added as covariates to control for individual differences. The model was significant for perceived musical control χ^2^ = 9.86, *p* < 0.05, and feeling in touch with the music χ^2^ = 19.90, *p* < 0.001. No effect was observed for mood χ^2^ = 3.79, *p* > 0.05.

A *post hoc* analysis was conducted to assess differences found between the three exploratory measures. Analysis revealed perceived creativity to be significantly lower in the music control only condition (M = 31.05, SD = 27.69) compared to music-feedback exercise (M = 44.04, SD = 25.85) and physical exercise with music listening (M = 59.94, SD = 24.88). Perceived musical control was found to be higher in the physical exercise with music listening condition (M = 61.72, SD = 24.17) compared to music control only (M = 31.85, SD = 27.69) and music-feedback exercise conditions (M = 44.05, SD = 25.85) (*p* < 0.05) but not different between music-feedback exercise and music control only conditions (*p* > 0.05).

## Discussion

We hypothesized that musical control would more strongly increase Divergent Thinking in participants than physical exercise with music listening and that the use of musical feedback in combination with physical exertion (music-feedback exercise) would have a stronger effect on Divergent Thinking capability in participants than either of the other two conditions (music control only without physical exertion using a knob to control the music and physical exercise with music listening). This experiment demonstrates that the music-feedback exercise condition significantly increased the participant’s scores in the Guilford Alternative Uses Task. This shows that the interaction between musical control and physical exertion can act as a driver for an increase in creativity. Surprisingly, no effects on Divergent Thinking were observed for the physical exercise with music listening and music control only conditions. In the present study, we employed a novel form of music for the feedback used in the interventions. This style of music was intentionally left to be vague and difficult to interpret to introduce a variability between participants in how much they felt they could be in control of making the music. As such, this style of music has, as of yet, not been used in other experiments with music-feedback exercise. This may act as a confounding variable to some of our results such as perceived creativity, agency, and mood effects.

A cognitive process that has previously been shown to influence Divergent Thinking is mood state, with positive mood increasing Divergent Thinking (Ritter and Ferguson, 2017). We were surprised to see that mood had no effect on Divergent Thinking scores in the present study. This seems to indicate that mood is not the only driver of Divergent Thinking effects in the current paradigm.

**FIGURE 2:**
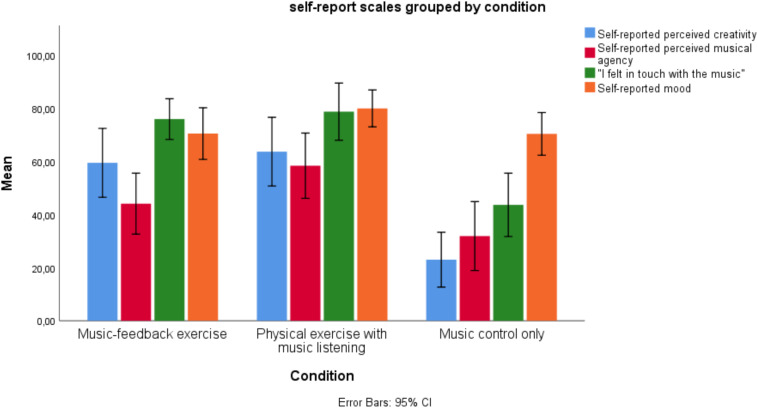
Mean results of self-report scales collected postintervention between conditions. Error bars represent 95% confidence interval.

Participant questionnaire responses following both physical exercise interventions showed increased confidence in one’s own current creative ability compared to music control only (Figure 2). However, the test of the actual Divergent Thinking performance showed a discrepancy of perceived creative ability and actual performance in the condition where participants performed exercise without making music, such that the actual performance was lower than subjectively believed. The condition that combined making music and making exercise (music-feedback exercise/Jymmin^TM^) resulted in a strongly enhanced Divergent Thinking capability that seems to be an interaction effect of making music and exercising. This effect of physiological arousal on perceived creativity suggests a discrepancy between believed and actual abilities for generating novel and useful ideas. This could relate to a perceived relation between effort and outcome such that, if one engages in a greater physical effort, one expects a greater creative outcome (which was, however, only true for the music-feedback exercise condition but not the physical exercise with music listening condition).

Previous evidence has shown that Jymmin^TM^ can more strongly increase mood (Fritz et al., 2013b), which probably relates to findings that, postintervention, it leads to increased pain threshold (Fritz et al., 2018) and decreased perceived exertion after physical exercise (Fritz et al., 2013a). This probably relates to endorphin release (Fritz et al., 2018). In the current experiment, we did not focus on investigating mood effects of the intervention and only assessed this using a single-item visual analog scale. This measure is not as sensitive as a validated mood assessment tool such as the MDMQ (Steyer et al., 1994). It is furthermore possible that the assessment of mood after the administration of the Guilford’s Alternative Uses Task is responsible for the absence of an observed mood effect. Future research should investigate the transient nature of mood effects as discussed in Fritz et al. (2013b). It is, however, also possible that individual differences across groups are affecting the data, as we did not collect mood data before the intervention. Furthermore, the music feedback used in the current approach was different than in the previous experiment and chosen with the aim to introduce a high variability between participants in how much they feel in control of the music. It is, at present, uncertain whether we have managed to capture the level of perceived musical control using only a single item on a questionnaire. A complete questionnaire, devoted to mapping out perceived musical control, would have allowed us to make a stronger argument in this regard.

It is still uncertain if the quality of the music stimulus, e.g., a higher level of complexity and challenge to create an audiomotor mapping with the musical output signal, modulates the level of Divergent Thinking capability. Given how physical exercise in isolation has previously been shown to enhance Divergent Thinking capabilities (Colzato et al., 2013), we expected to see an increase in Divergent Thinking capabilities in the physical exercise with music listening condition. Such an effect was not observed. It is unclear why this was the case. It may relate to the style of music used in the current experiment that participants were additionally passively listening to. We did not observe an increase in perceived musical control in the music-feedback exercise condition, probably because the mapping of action to sound feedback was intentionally composed to be difficult to understand to investigate an influence of this agency experience on the amplitude of other effects.

### Application in Sports

Athletes in several sports (e.g., team sports) benefit from increased creativity in terms of Divergent Thinking abilities to, e.g., envision unexpected actions to “out-play” the opponent(s). Examples may include quickly generating ideas for how to pass a ball through the opposing team and generating novel spatial paths for where to send the ball in as few strokes as possible in a golf setting. It has been argued that the capability to be creative about both one’s own and an opponent’s action capabilities in real time is a crucial component to optimal performance in fast, dynamic sports including a number of team sports such as soccer, basketball, and football (Fajen et al., 2009; Craig and Watson, 2011). The current findings suggest that athletes could benefit from interventions involving music-feedback exercise in a warm-up procedure before a training or competition. Given how athletes already rely on other physical exercises as their warm-up procedure, it would not be a difficult transition to switch to a procedure that also enhances Divergent Thinking while still performing the function as a warm-up procedure. Accordingly, music-feedback exercise (Jymmin^TM^) may be well suited to be part of a warm-up procedure that also aims toward optimizing creative performance during sports.

Music-feedback exercise may also increase motivation in sports contexts. It has previously been shown that creativity has the potential to increase the ability to find motivation (Joy and Breed, 2012). The association between creativity and motivation is not yet fully established; however, we speculate that increased idea generation would reinforce motivation by generating novel reasons for why to not give up. While this effect may swing both ways in the normal population by allowing for increased reasoning both for and against the exercise, athletes are already motivated by default due to an innate positivity bias and are thus probably more likely to generate motivational ideas. We suggest that music-feedback exercise may therefore be used as a form of especially encouraging regeneration training where athletes train with reduced intensity with the aim of recuperating before another intensive bout of exercise.

### Further Limitations and Future Study

The current study does not investigate if effects of music-feedback exercise to some degree depend on the quality of musical feedback experienced by the users. This has not been systematically addressed and should be done so in a future study. In the current discussion we argue that an increase in Divergent Thinking following a specifically designed warm-up procedure would lead to an increase in sports performance. This is not directly addressed in the current study. Future research should examine the relationship between music-feedback exercise and sports performance. It is also possible that intraindividual factors such as intelligence and music preference may have contributed to the observed results. We did not assess musical proficiency or preference, which may also have had an effect on the processing of the rather experimental music. We focused on musical control as opposed to music listening. In order to keep the conditions as similar as possible, we introduced passive music listening to the physical exercise condition. This was mainly to eliminate acoustics as a potentially confounding variable. Although the music remained a constant across conditions, it is still possible that this has acted as a confounding variable. Future studies on the topic should include a control condition devoid of both music and physical exercise. Interesting future directions to further investigate the effects reported in the current study would be to assess how increasing Divergent Thinking capability through a musical intervention would relate to tolerance to uncertainty and adaptive coping behavior, which both is highly relevant in rehabilitation and education.

## Conclusion

In conclusion, the current data demonstrate an increase in Divergent Thinking capabilities as an interaction between musical control and physical exercise. We discussed how an intervention that combines musical control with physical exercise (Jymmin^TM^) could help with creative blocks. We also discuss how this intervention may further be beneficial to athletes as part of a sports warm-up routine before training or competition and as part of regeneration training.

## Data Availability Statement

The raw data supporting the conclusions of this article will be made available by the authors, without undue reservation.

## Ethics Statement

The studies involving human participants were reviewed and approved by the Ethics Committee of the University of Leipzig. The patients/participants provided their written informed consent to participate in this study.

## Author Contributions

TF, LS, EB, and AV designed the study. TF, LS, and MM analyzed the data. TF, MM, and LS wrote the manuscript. All authors contributed to the article and approved the submitted version.

## Conflict of Interest

Jymmin^TM^ is a spin-off of the Max Planck Society (MPS). MPS and TF are shareholders in the Jymmin GmbH Company. The remaining authors declare that the research was conducted in the absence of any commercial or financial relationships that could be construed as a potential conflict of interest.
